# Tackling NCDs: The Need to Address Alcohol Industry Interference and Policy Incoherence Across Sectors Comment on "Towards Preventing and Managing Conflict of Interest in Nutrition Policy? An Analysis of Submissions to a Consultation on a Draft WHO Tool"

**DOI:** 10.34172/ijhpm.2020.172

**Published:** 2020-09-06

**Authors:** Belinda Townsend, Mia Miller, Deborah Gleeson

**Affiliations:** ^1^Menzies Centre for Health Governance, School of Regulation and Global Governance, Australian National University, Canberra, ACT, Australia.; ^2^Centre for Alcohol Policy Research, School of Psychology & Public Health, La Trobe University, Melbourne, VIC, Australia.; ^3^School of Psychology and Public Health, La Trobe University, Melbourne, VIC, Australia.

**Keywords:** Health Policy, Conflict of Interest, Alcohol Industry, Trade Policy, Non-communicable Disease, Australia

## Abstract

Ralston et al highlight the ways that different actors in global nutrition governance conceptualise and frame the role of non-state actors in governance arrangements, including the potential for conflict of interest (COI) to undermine global health efforts. The authors argue that the World Health Organization (WHO) draft tool on managing COI in nutrition policy is an important innovation in global health, but that further research and refinement is needed for operationalising the management of COI with diverse actors in diverse contexts. In this commentary, reflecting on strategic framing and industry interference in policy-making, we argue for the urgent need for states and intergovernmental organisations to prevent alcohol industry interference in the development of national and global alcohol policy. We argue that policy incoherence remains a key barrier, where governments pursue health goals in the health sector while pursuing exports and market liberalisation of health harmful commodities in the trade sector.

Ralston and colleagues^1^ address an important issue in global health, that of how to manage non-state actors’ engagement with governments and intergovernmental organisations in ways that do not undermine or interfere with their mandates to promote public health. The need to develop mechanisms and tools to prevent industry interference that weakens public policy-making for health is important for all areas of government. It is particularly in focus in the area of non-communicable diseases (NCDs) which now account for more than 70% of preventable deaths globally. The big three harmful commodity industries – tobacco, alcohol, and ultra-processed and unhealthy foods - are key risk factors for NCDs. As Ralston et al^1^ note, these industries are treated differently in global health. The World Health Organization (WHO) Framework Convention on Tobacco Control (FCTC), which came into force in 2005, is a novel global health treaty which provides a range of measures for the reduction of tobacco consumption. While the treaty text does not explicitly use the term ‘conflict of interest’ (COI) it does include the clear requirement that governments should not engage with the tobacco industry in the development of public health policies:

*“In setting and implementing their public health policies with respect to tobacco control, Parties shall act to protect these policies from commercial and other vested interests of the tobacco industry in accordance with national law.”* (Article 5.3)

 The question of engagement between WHO and other industry and non-state actors was in focus in the recent WHO reform process which led to the development of a new WHO Framework of Engagement for Non-State Actors, which defines an institutional COI as:

*“A situation where WHO’s primary interest as reflected in its Constitution may be unduly influenced by the conflicting interest of a non-State actor in a way that affects, or may reasonably be perceived to affect, the independence and objectivity of WHO’s work”* (para 24).

Within this framework, the potential risk of institutional conflicts of interest “could be the highest in situations where the interest of non-State actors, in particular economic, commercial or financial, are in conflict with WHO’s public health policies, constitutional mandate and interests” (para 26). As Ralston et al^1^ note, it is within this context that the WHO draft tool on preventing and managing COI in nutrition policy was developed as guidance for Member States.

 In this commentary, we examine the issue of COI in regards to the alcohol industry. Drawing on the WHO’s definition cited above, we argue that the alcohol industry has an institutional COI in public health because its objectives for increasing consumption of alcohol products conflict with a public health mandate to reduce alcohol harm. We focus in particular on the industry’s use of strategic framing, drawing parallels to Ralston’s study of frames of COI in global nutrition policy. We illustrate the case of Australia where attempts to introduce strong COI for the alcohol industry recently were watered down. Furthermore, we show that incoherence between government trade and health portfolios remains a key barrier for NCD policy, requiring the prioritisation of health and well-being in a whole of government approach.

## The Need to Prevent Alcohol Industry Interference in Global and National Health Policies

The alcohol industry has a well-documented suite of strategies that it uses to influence global and national health policy-making.^2^ A recent systematic review, for example, found several short-term and long-term strategies used by industry actors which led to policy influence including a weakening or reversal of proposed public health regulations.^2^

One example of influence at the global level is though forming partnerships with legitimate global health bodies. The Global Fund to Fight HIV/AIDS, Tuberculosis and Malaria has come under significant global health criticism for sponsoring multinational alcohol companies’ projects in low and middle income countries.^3^ As Matzopolous et al^3^ note, potential conflicts can arise for global health bodies in such partnerships “in that the industry can appear to be dealing with the social responsibility requirement of addressing the harms associated with its products – in itself a form of advertising – with an intervention that does not reduce availability of alcohol or consumption of its products.” Strong opposition from civil society and some member state donors has forced some of these partnerships to cease, the most recent being the Global Fund’s suspension of a partnership with the multinational beer company Heineken.^4^

In recent global health and alcohol policy discussions, experts have argued that Member State inaction on the *WHO Global Strategy to Reduce the Harmful Use of Alcohol*requires focused discussions on the potential for a globally binding instrument like the FCTC, and clear rules and guidance for preventing alcohol industry interference.^5^ At the request of Member States in February 2020, the WHO is presently developing an action plan (2022-2030) to effectively implement the Global Strategy. Public health advocacy organisations and non-government organisations have praised this move to accelerate action on reducing alcohol harm, but have continued to call for a strong focus in any plan to prevent alcohol industry interference.^6^ As the NCD Alliance^7^ has argued: “*It is important that the Action Plan addresses industry interference as a barrier to alcohol policy progress… It is paramount that the alcohol industry are not allowed to dilute the enormous potential of the forthcoming Action Plan to save lives from their products.*”

This problem of alcohol industry interference is mirrored at the national level. In Australia, for example, attempts to introduce strong guidance on preventing alcohol industry interference appear to have been weakened through the recent iteration of the National Alcohol Strategy. In 2017, the Australian Federal Government released a draft strategy document to introduce a new strategy following the expiration of the last National Alcohol Strategy in 2011. This draft included statements that “Australia does not support any ongoing role for industry in setting or developing national alcohol policy” (p. 24).^8^ This was a significant indication of strong opposition to alcohol industry interference and arguably went further than the WHO draft nutrition COI tool by indicating exclusion of the alcohol industry from health policy-making (much like the WHO FTCT and tobacco). However, following consultations with stakeholders including industry actors, the only remaining references to the alcohol industry in the final 2019 Strategy document are favourable towards the industry; that the “alcohol manufacturing industry, wider retail and hospitality industries, advertising, broadcasting and sporting industries play a significant role in Australia’s economy and social fabric” (p. 3)^9^ and that “industry, local businesses, community groups and individuals can also take action in reducing alcohol related harms” (p. 13).^9^ This apparent shift in framing indicates a move away from acknowledging the alcohol industry as having a COI in public health policy-making, to promoting the industry as a partner for economic growth, employment and society.

## Framing Engagement

This apparent shift in framing in the Australian approach to the alcohol industry mirrors competing frames found in Ralston and colleagues’^1^ study of the nutrition sphere. Ralston et al identify two broad frames; on the one hand a “collaboration and partnership frame” promoted by most commercial actors and some states that endorses multi-stakeholder approaches and plays down potential conflicts of interest; and on the other a “restricted engagement frame” held by public health advocacy groups and some international agencies that emphasises core tensions between public health and commercial actors. Framing analysis usefully reveals the underlying assumptions and narrative tools that policy actors use to advance their objectives.^9^

Ralston et al show that commercial actors largely framed the WHO draft tool as “inappropriate, unworkable and incompatible with the Sustainable Development Goals” and “viewed it as unduly restricting scope for private sector engagement in nutrition policy.” Interestingly, commercial actors frequently invoked a “claimed disjuncture” between the WHO draft tool and the United Nations Sustainable Development Goals’ focus on multi-sectoral and partnership approaches (p. 5).^1^ This strategic framing appears to serve as an attempt to discredit the WHO as the forum for deliberations on non-state actor engagement, and instead to “forum shift” the debates around engagement to a more partnership-friendly platform though United Nations partnerships. Other framing rhetoric identified by Ralston et al (p. 4)^1^ included rejection of any comparison between the food industry and tobacco industry, “anti-exclusionary” framing of the tool as inherently distrustful of industry, claiming that existing practices were satisfactory and there was therefore no need for the tool, and arguing that extensive industry engagement was compatible with good governance. Analysis of alcohol industry submissions made to the Australian Government on the draft National Alcohol Strategy also included anti-exclusion framing and argued for the need to engage ‘whole-of-community’ including industry.^11^ Other arguments by the alcohol industry included; that there was no urgent need for a strategy as existing practices were satisfactory; a strong preference for voluntary self-regulation; claims of the health benefits of moderate alcohol consumption; and arguments disputing evidence put forward eg, disputing the causal link between alcohol advertising and alcohol consumption.^11^ These frames appear to have been invoked to counter public health attempts to introduce robust guidance on preventing industry interference in Australia. It is noteworthy that the final document reflected the alcohol industry’s preferred framing of its engagement in policy-making.

## Managing Incoherence Beyond the Health Sector

 Finally, discussions about public health engagement with non-state actors are only part of a wider problem of policy incoherence for NCDs which goes beyond the health sector. As Ralston et al note, the WHO draft tool on COI for nutrition policy is intended to be used by Ministries of Health to evaluate whether and how to engage with non-state actors. Outside Ministries of Health, however, government departments of trade and investment seek to expand market liberalisation and export of commodities, where alcohol, tobacco and ultra-processed and unhealthy foods are often treated like any other commodity.

In particular, trade and investment agreements negotiated between countries can serve as structural drivers for NCD risk factors. For example, an analysis of preferential trade agreements between Australia and sixteen other countries found a statistically significant increase in the share of Australian alcohol beverage imports in trade partner countries, with a larger effect in countries with a lower rate of consumption.^12^ This points to an incoherence facilitated by a deeper neoliberal paradigm which prioritises industry objectives and market liberalisation, “enshrining institutional mechanisms that have given rise to existing systems of governance of product environments… [and] creating structural barriers to the introduction of meaningful policy action to prevent NCDs.”^13^ For example, the Comprehensive and Progressive Agreement on Trans-Pacific Partnership negotiated between fifteen Pacific Rim countries (following the US withdrawal in 2017) contains an annex on supplementary labelling in relation to wine and distilled spirits (Annex 8A). Public health concerns regarding the annex include whether it could be invoked in ways that restrict the capacity of governments to introduce public health labelling laws.^14^

Interviews with key stakeholders in trade policy in Australia have shown a strong ideational view amongst government officials and some politicians that trade agreements are for expanding markets and “for industry.”^15^ As one informant reported; “in the TPP we did not get far on alcohol labelling because DFAT [Department of Foreign Affairs and Trade] saw market access … as being more important than a public health principle.”^15^ Managing COI and industry engagement in health policy is thus only part of a larger problem of incoherence across government sectors. In the case of trade and investment policy, the use of health impact assessments before, during and after trade agreements are signed is one mechanism that could enable the prioritisation of potential health impacts of trade deals.

 COIs are as rife in alcohol policy as they are in nutrition policy. Addressing them will require identifying and counteracting the strategic framing used by the alcohol industry, preventing industry interference in national and global health policy, and actively promoting health and well-being in other sectors to manage policy incoherence.

## Ethical issues

 Not applicable.

## Competing interests

 DG reports they are the convenor of the Political Economy of Health Special Interest Group of the Public Health Association of Australia (PHAA) and often represents the PHAA on matters related to trade agreements and public health. BT reports they are a member of the PHAA and sometimes represents the PHAA on matters relating to trade and public health.

## Authors’ contributions

 BT wrote the first draft and discussed with MM and DG. All authors approved the final text.

## Authors’ affiliations

^1^Menzies Centre for Health Governance, School of Regulation and Global Governance, Australian National University, Canberra, ACT, Australia.^2^Centre for Alcohol Policy Research, School of Psychology & Public Health, La Trobe University, Melbourne, VIC, Australia.^3^School of Psychology and Public Health, La Trobe University, Melbourne, VIC, Australia.
